# Enzymes for Leather Processing: Effect on Pickling and Chroming

**DOI:** 10.3390/ma14061480

**Published:** 2021-03-17

**Authors:** Renata Biškauskaitė, Violeta Valeikienė, Virgilijus Valeika

**Affiliations:** Faculty of Chemical Technology, Kaunas University of Technology, Radvilenu pl. 19, 50254 Kaunas, Lithuania; renata.biskauskaite@ktu.lt (R.B.); violeta.valeikiene@ktu.lt (V.V.)

**Keywords:** hide, enzyme, collagen, pickling, chroming, leather

## Abstract

Recently, increasing attention has been paid to the application of enzymes in a wide variety of leather production processes. The aim of the present study was to investigate the action of enzymatic pickling on derma’s collagen and the influence of this action on subsequent processes and properties of chromed and finished leather. The application of active in acidic medium proteolytic enzymes in the pickling process led to an additional impact on derma structure: collagen was more strongly affected and the porosity of the pelt dermis was reduced, but the hide became more thermally stable. The enzymatically pickled pelt bonded more chromium and reached higher shrinkage temperature while chroming; dyes penetrated deeper; such leather bonded more fatliquors. On the other hand, the action of enzymes worsened the physical–mechanical properties of the leather, as the experimental leather was weaker than the conventional one. The first was characterised by weaker grain layer and had significantly higher relative elongation. Therefore, as some properties improve and others worsen during such a process, the application of every enzyme should be carefully investigated and optimized to produce a leather with defined properties.

## 1. Introduction

Enzymes are gaining more recognition because of advancements made in purification, development and improvement, and they are also considered environmentally friendly. Due to a growing interest in environmentally safer processes, the leather industry has started using the concept of cleaner production to minimize its impact and reduce the loss of chemicals, water and raw materials. The use of enzymes for leather processing has become a very efficient tool in achieving these goals. Another additional, but no less important, benefit of replacing harmful substances by enzymes is the resulting products of better quality.

Currently, vast efforts are being made to apply enzymes in a wide variety of leather production processes. For example, none of leather technologists can imagine a bating of leather without use of enzymes as enzymatic bating has become the classical method. There are two main reasons allowing the wide employment of enzymes in the leather manufacture:

1. The main object of processing is protein-collagen, which is sensitive to the action of enzymes. Despite the fact that during the processes, the structure of collagen changes to become even highly modified, thermally and bacteriologically resistant, it still remains a protein that can be affected by a specific enzyme under specific conditions.
2. The fast-growing enzyme industry and the diversity of products it provides.

Enzymes can be used in the first step of skin treatment, i.e., soaking of skins/hides. Various enzymes can be employed as soaking auxiliaries [1]: proteases, lipases, amylases, chondroitinases, amidases, phospholipases, etc. The identified advantages of using enzymes for soaking include faster rehydration and removal of interfibriliary materials [2]. The action of enzymes is one of the methods that leather technologists use just to accelerate soaking and minimize the use of harmful unhairing chemicals [3,4,5].

On the other hand, a possibility of side effects must be very reasonably estimated before applying enzymes in leather processing. Xu et al. [6] explored commercial proteases widely used in soaking, unhairing, liming and bating, and concluded that care was essential in selecting proteases for leather-making processes to avoid grain loosening and damage from the excessive removal of elastin.

The increase in the amount of enzyme in a soaking solution affects faster rehydration, higher content of removed non-collagen proteins but, unfortunately, has an effect on the collagen of hide not only during the soaking but also during subsequent liming as well, which can be the reason for defects in the finished leather [7].

The next step in employing enzymes is the degreasing of hides/skins. The degreasing is generally applied to raw materials with a high fat content in skin tissues: pig, sheep skins, among others [8,9]. For an enzymatic preparation to be an effective degreasant, it needs to perform a triple action of proteolysis, lipolysis and emulsification [10,11]. The enzymatic degreasing can be carried out as, for example, during skin soaking or simultaneously with other processes: soaking, liming, bating and de-pickling, after which optimum degreasing treatment is realized [12].

Conventionally, the hide/skin unhairing stage employs lime and sulphides and, therefore, the process is considered to be the most polluting one in leather processing. In addition, the purification of unhairing solutions remains very difficult and expensive. Therefore, the application of enzymes as environmentally friendly unhairing auxiliaries is a very attractive alternative. Khambhaty [13] has mentioned that about 70 unhairing proteases/keratinases from bacteria and fungi indigenously isolated and reported by various researchers.

There are a few possible ways to adopt the enzymes for the unhairing process. The first one is when the enzymes are applied for a pure enzymatic process, in such a case, the unhairing effect is achieved, owing to the enzyme used [14,15,16]. Unfortunately, so far, such an unhairing method has been applied merely on laboratory scale [13].

The next way is to use enzyme and oxidising agents. In such a case, enzymes are used for the main unhairing and oxidisers are used for degrading of hair remnants. The proteases acting in alkaline or acidic medium can be applied in such cases [17,18]. It should be noted that the oxidative–enzymatic unhairing process has also not been adopted in practise.

As Fennen et al. have reported [19], proteolytic enzymes can accelerate unhairing in the liming step. A reliable unhairing process with low sulphide and lime content can provide an excellent unhairing effect and lead to a substantial reduction in the load to the environment of sulphide, nitrogen, COD, and sludge on the environment [19].

In conventional leather processing technologies, the bating process is based on the use of proteases in alkaline medium. The main aim of bating is to break down the non-structural proteins of skin/hide, such as albumin, globulin and mucoid or cementing substances, eventually facilitating the splitting up of collagen fibres, so as to help in the penetration of tanning materials, thereby giving the finished leather the desired feel, softness, pliability, elasticity and other characteristic properties [1,20].

New developments in this field include the use of enzymatic products that allow for an execution of bating, even at acidic pH. Yongquan [21] reported that acid protease, from *Aspergillus usamii*, has been found to be a more effective bating agent for sheep pelt than neutral protease. In another study, the bating of pelts after using peracetic acid for de-liming was studied and the use of enzyme preparations active in acidic medium was suggested for the bating of such pelt [22].

Chrome tanning is the most important method to obtain leather of high thermal and bacterial resistance. Enzymes are not directly involved at this stage; on the other hand, the enzymatic treatment in previous stages influences the quality of leather [23].

It should be noted that investigation of the possibility to use enzymes do not terminate at this process. The attempts have been made to employ enzymes for processes following tanning z such as re-tanning, dyeing, and even fatliquoring.

Kanth et al. reported [24,25] that the application of enzymes for vegetable tanning process without pickling has resulted in more than 97% tannin exhaustion in the case of the experimental process, an increase of 12% compared with the conventional vegetable tanning process.

A post-tanning enzymatic treatment changes the properties of the leather and thus influences the subsequent processes. The study of Song et al. [26] has shown that leather treated with protease flavourzyme showed improved fastness properties against rubbing and dry cleaning compared with untreated leather. The use of enzymes directly in leather dyeing has led to better exhaustion of dye as collagenase-assisted process allows reaching 99% uptake of dye [27]. Rameshkannan et al. adopted horseradish peroxidase in fatliquoring process to decrease amount of alkyl phenol ethoxylates in leather [28].

However, more research should be carried out to study the enzyme effect and application in other leather industry processes. The emergence of commercial proteases that are active in acidic media has made it possible to use them directly in the skin/hide pickling process. Such use of enzymes might have an additional effect on collagen, which in turn would change its behaviour during further processes. Accordingly, the aim of the presented research was to study an action of proteolytic enzyme preparations on derma during the pickling process and to assess how the effect reflects on the properties of chromed and finished leather.

## 2. Materials and Methods

### 2.1. Raw Material

Bovine hide cured by salting (bull hide; 26 kg; purchased in Kėdainių Oda, Kėdainiai, Lithuania) was cut into pieces (15 × 15) cm and series of samples were prepared from these pieces. The series were formed in such a way that samples from all hide parts would be presented in each series.

The pieces from the rump part were marked and, afterwards, used for FTIR analysis and for the determination of qualitative indexes of hide and leather, such as porosity, amount of chromium, shrinkage temperature, strength properties, etc.

### 2.2. Chemical Materials

The chemicals used for the analysis were of analytical grade. The analytical and technical grade materials were used for the technological processes.

Enzyme preparations (EP) of Zime SB (River Chimica, Ponte a Egola, Italy), which is the bating enzyme for acid bate, and Novo Bate WB, isolated from Bacillus microorganisms (Novo Nordisk, Bagsvaerd, Denmark), which is commonly applied in the re-bating process of wet-blue stock, were used for the pickling process execution.

Other technical products used for the technological processes were: Cromeco 33 Extra, a basic chromium sulfate (contains 25% of chromium (III) oxide, 33% basicity) produced by Gruppo Chimico Dalton (Limbiate, Italy). Neutragene MG-120 ((for increasing the chromium compounds’ basicity) and fatliqours as Oleal 146, Oleal 1946, Fospholiker 661 and Fospholiker 6146, which are technical products produced by Codyeco S.p.a. (Vicolo del Grano, Italy). Dye Sellaset red H, which is produced by TFL Ledertechnik GmbH (Rheinfelden, Germany).

### 2.3. Technological Processes

All series of samples from soaking until pickling were processed according to conventional leather processing technology: soaking, fleshing, sulphide liming-unhairing, and deliming-bating were carried out. The processing of pieces with chemicals was performed in a laboratory drum with a capacity of 3 litres.

Pickling, chroming and post-tanning processes were executed as follows:

Pickling (% are based on limed hide mass): water 80%; temperature 20–22 °C, NaCl 5.5%, 15 min; NaHCOO 1%, 20 min; H_2_SO_4_ 0.5%, 15 min; H_2_SO_4_ 0.5%, 15 min; H_2_SO_4_ 0.5%, 1 h; EP Novo Bate WB 0.5; 1 or 2%, or EP Zime SB 1, 2 or 3% (control without EP), 4 h.

Chroming (in pickling solution): temperature 20–22 °C, Chromeco 33 Extra 6%, 20 h; Neutragene MG-120 0.35%, 2 h, water 100%; temperature 40–42 °C; 2 h. Drain.

Washing (here and for subsequent processes % are based on chromed leather mass): water 100%, temperature 40–42 °C, 1 h. Drain.

Neutralization: water 150%; temperature 35–40 °C; NaHCO_3_ 1.5%; 0.5 h; NaHCOO 2.0%; 1.5 h. Drain.

Washing: water 100%; temperature 40–45 °C; 0.5 h. Drain.

Dyeing: water 200%; temperature 58–62 °C; dye Sellaset red H—1.5%, 1 h. Drain.

Fatliquoring: water 200%; temperature 58–62 °C; Oleal 146 2%; Oleal 1946 4%; Fospholiker 661 3%; Fospholiker 6146 4%; 1 h; HCOOH 0.5%; 20 min; HCOOH 0.5%; 20 min. Drain.

Washing: water 100%; temperature 30 °C; 0.5 h. Drain.

Drying: the samples were placed on a table and dried in a free state for 48 h at 22–25 °C.

Notes: The percentage amounts of materials were based on limed hide (pickling-chroming) or chromed hide (post-tanning processes) weight. Regime for all processes: run continuously.

### 2.4. Analysis Methods

A proteolytic activity of EP was determined using the Anson method [29]. Sodium caseinate was used as a substrate.

The number of collagen proteins removed was estimated from the amount of hydroxyproline in the pickling solution using a photo-colorimetric method [30]. The samples of the pickling solution after the process were hydrolysed using HCl at 120 °C for 10–12 h. A formation of coloured soluble product was based on reaction of hydroxyproline with *p*-dimethylaminobenzaldehyde. The absorption was measured with a spectrophotometer GENESYS-8 (Spectronic Instruments, Cheshire, UK) at 558 nm wavelength.

Chromium compound exhaustion was estimated by determining the concentration of chromium in the initial chroming solution and in a mixture of used chroming solution and washing (after chroming) solution. The concentration of chromium in solution was determined according to the method described in the literature [31]. The method involves the oxidation of the chromium presented in the solution into hexavalent state using hydrogen peroxide, and analysis of the solution by iodometric titration.

The porosity of the hide was determined according to the method described in the literature [31]. Dehydrated with acetone leather samples were cut into regular rectangles of 4 cm × 5 cm and kept under standard conditions at a temperature of 20 °C and a humidity of 52% for 24 h. The length and width of the samples at three points and the thickness of the sample at least at nine points were measured using a micrometer (Mitutoyo, Kawasaki, Japan). Based on the average values of these measurements, volume of the sample was calculated and the sample was weighted on precision balances. The sample was then immersed in a low-volatile solvent of known density and kept under vacuum (0.01 MPa) for 10 min. The sample was taken out, the excess solvent was removed, and the sample was weighted again. The volume of solvent absorbed corresponds to the volume of the pores. Porosity is the ratio of pores to sample volumes expressed as a percentage.

Samples of hide chosen for IR-spectroscopic analysis were split to obtain a 0.9–1.0 mm width of the upper layer. The samples for the IR-spectroscopic analyses were prepared as pellets using 200 mg of optically pure KBr and 2 mg of hide tissue, which was taken from the surface formed after splitting. An infrared reflection spectrum was obtained using a Perkin-Elmer FTIR Spectrum GX (Waltham, MA, USA) spectrometer. The resolution was 1 cm^−1^, scan rate 0.2 cm/s and scan number 16 times. Before the evaluation of porosity and IR-spectroscopy analysis, the hide samples were dehydrated with acetone [32].

The shrinkage temperature of the hide was measured according to the standard [33]. The shrinkage temperature of the chromed leather samples was determined as described in the literature using special equipment and replacing the distilled water with glycerol [31].

The penetration of dyes through hide tissue was measured using the special optical microscope with scale (magnification 15 times) MPB-2 (Izyum Instrument Making Plant, Izyum, Ukraine).

The strength properties, the amount of chrome compounds in the leather, matter soluble in dichloromethane, and volatile matter were determined according to the standards [34,35,36].

### 2.5. Statistical Analysis

All data were expressed as the average value of triplicate measurements. One sample was used for one measurement. Standard deviations did not exceed 5% for the values obtained.

## 3. Results and Discussion

**Figure 1:**
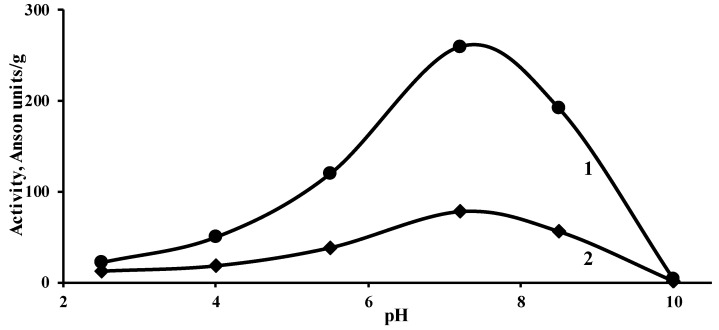
Dependence of EP Novo Bate WB (**1**) and EP Zime SB (**2**) activity on pH of medium (temperature 20 °C; duration of action 20 min).

A pickling process begins at relatively low pH and it is necessary to know the possibilities of enzyme preparation (EP) to act in such medium. Accordingly, the first step of the experiment was to elucidate the dependence of EP activity on the pH of the medium (Figure 1).

Despite the fact that the activity of the two EPs is very different, the trend of activity as a function of pH is very similar: they are most active at pH 7.2; become almost inactive at pH 10; have close activity values at pH 2.5.

Since at the beginning of the process, the pH value of the pickling solution was significantly lower than 2.5, the EP were added (EP Novo Bate WB 0.5; 1 or 2% (variants 1; 2 and 3); EP Zime SB 1; 2 or 3%; (variants 4; 5 and 6)) after 1 h since the last portion of sulphuric acid was poured when the pH of the pickling solution reached pH not less than 2–2.5. After the addition of EP, the pickling was continued for 4 h. Control pickling was carried out without the addition of EP (variant 7). The total process duration was the same in all cases.

**Figure 2:**
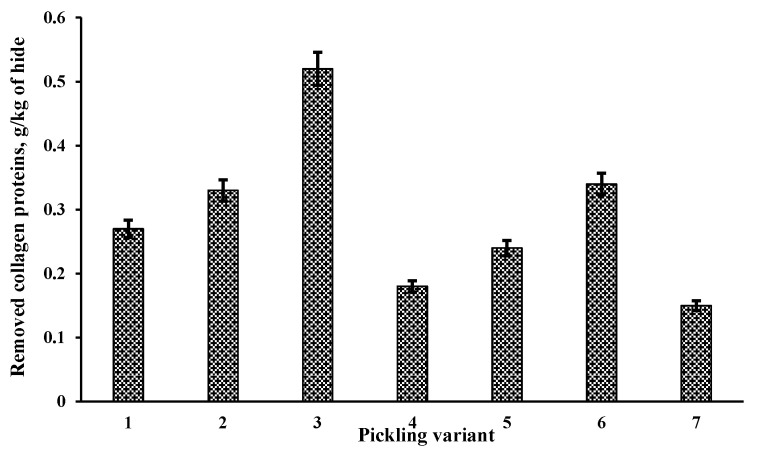
Effect of pickling method on collagen.

After the pickling, the influence of the process on pickled pelt was assessed by determining the amount of removed collagen proteins during the pickling (Figure 2), shrinkage temperature and porosity of hide (Table 1). The addition of EP had an effect on the collagen of the treated hide. The number of removed collagen proteins increased 1.2–3.4 times. Usually, the amount of collagenous proteins removed during conventional liming-sulphide unhairing varies in the range of 0.2–0.5 g/kg [37,38], therefore the enzymatic pickling can be considered as the process significantly acting on a hide collagen.

There was also a noticeable effect not only on collagen as a separate hide protein, but also on the overall structure of the dermis. During the action of enzymes, intense changes of the dermis took place leading to a decrease in the porosity of the hide dermis: accordingly, it decreased more when the action of enzymes was stronger (Table 1). The decrease in porosity resulted in a higher value of the shrinkage temperature [39]. Such results can be explained by the possibility that small structural elements are opened up and approach each other during the enzymatic pickling.

It is obvious that the use of enzymes in pickling is not an end in itself, since it is important to evaluate how such a pickling method affects other processes and leather properties. Since pickling is the process that prepares a pelt for tanning, the next step taken was investigation of the chroming following the enzymatic pickling. Such indexes as chromium exhaustion during the chroming, content of chromium compounds in leather, its shrinkage temperature, and porosity were determined (Table 2).

Evidently, the pickled pelts treated with enzymes behave differently during chroming process. Probably, the least change was observed when evaluating the porosity results. The porosity of variously pickled samples changed just slightly—it increased or decreased, but no systematic nature of those changes can be observed. On the other hand, the dependence of porosity on the level of the influence of enzyme remained even after chroming: the samples affected stronger during the pickling were characterised by lower porosity after chroming.

Pickling itself is one of the pre-tanning operations in leather processing, which is carried out for conditioning skins prior to tanning [40]. The main function of pickling is to acidify the collagen. Therefore, the reactivity is modified because the chrome tanning reaction involves only ionised carboxyl groups [1]. Of note, Covington [1] mentioned another aspect of the pickling reaction, i.e., the contribution to opening up. For a majority of the processes, in conventional pickling, this reaction is unimportant because it is relatively slow [1]. However, the situation changes when enzymes are used in pickling. The reaction of opening up becomes important. The opening up of hide derma takes place during such pickling and this, accordingly, has influence on chromium bonding with hide proteins and the thermostability of the chromed hide.

The obtained results allow for the assumption that, firstly, the action of enzymes results in the better exhaustion of chromium compounds and increases their content in the leather. Herewith, this influences the higher thermostability of the hide as well. Secondly, the level of enzyme effect has no linear influence on the indices being evaluated; this does not necessarily mean that stronger enzymatic action is equal to better chroming. As seen from the results presented in Figure 2, the effect on collagen was the strongest when Novo Bate WB 2% was added to the pickling. This effect seems to have been just too strong and, accordingly, after the chroming of this sample, its shrinkage temperature was lower compared to that of the sample treated with a lower amount of this EP.

On the other hand, the sort of enzyme is important as well. Each enzyme acts specifically, despite the fact that they can be characterized as enzymes with similar action; for example, proteases [41].

The comparison of EP Novo Bate WB and Zime SB in respect of chroming process and chromed leather properties has revealed that the use of EP Zime SB for enzymatic pickling was more useful. The hide, which was pickled by adding EP Zime SB, had good thermostability after the chroming process, the exhaustion of chromium compounds from the chroming solution and the amount of chromium in the leather was higher, compared with the control sample (Table 2).

**Figure 3:**
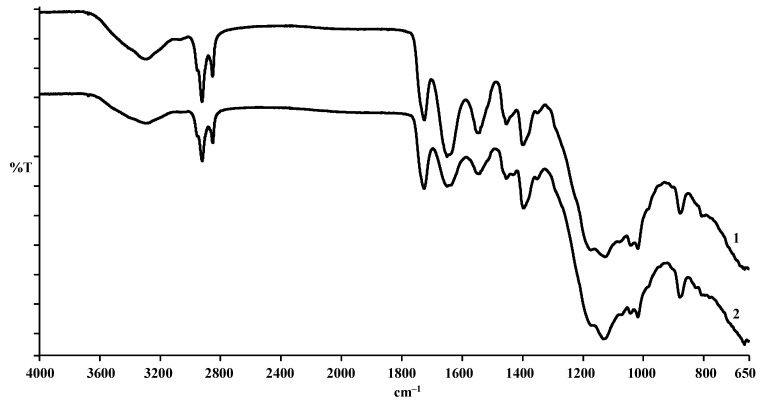
IR-spectra of hide. 1—pickled conventionally; 2—pickled adding EP Zime SB 3%.

In an attempt to evaluate how deep the effect of enzymes on the hide structure was, the pelt properties were assessed on the grounds of FTIR analysis. Therefore, IR-spectra after the chroming of the control leather sample and that pickled with the use of EP Zime SB 3% added were recorded and analysed (Figure 3).

The obtained IR spectra are almost identical (Figure 3). This means that even a comparatively strong effect of enzyme in pickling does not cause any observable effect on the supermolecular structure of the chromed leather, which can be reflected in IR-spectra.

**Figure 4:**
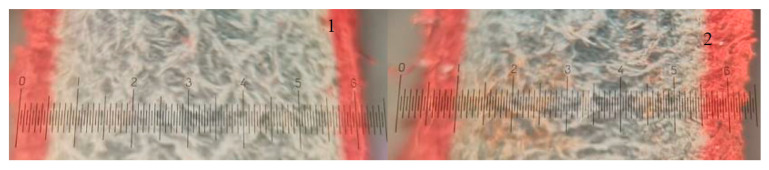
Images of dyed leather cross-section: magnification 15 times. **1**—pickled conventionally; **2**—pickled adding EP Zime SB 3%.

To evaluate the effect of enzymatic pickling on subsequent after chroming processes, the control leather sample and the sample pickled with the additive of EP Zime SB 3% were neutralised, dyed, fatliquored and dried. The consumption of dyes from dyeing bath, penetration of dyes through leather tissue, amount of matter soluble in dichloromethane (Table 3, Figure 4) and shrinkage temperature were determined and assessed after the processes.

The obtained results have shown that hide affected by enzymatic pickling behaved differently during the wet finishing processes in comparison with the hide pickled conventionally. The enzymatically treated leather absorbed more dye and the dye penetrated deeper into the leather tissue (Table 3). Of note, observing the images of dyed leather cross-sections (Figure 4) indicates that the penetration of dye through the grain layer improved significantly whereas just a slight improvement in penetration through the flesh layer could be observed.

Experimental leather absorbed more fatliquors as well. Overall, it seems that enzymatic treatment led to higher sorption of chemicals, i.e., chromium, dyes, and fatliquors. The leather properties dependable on the mentioned chemical materials improved as well. The shrinkage temperature of the experimental leather increased, as did content of chromium and fatliquors. Dyes also penetrated deeper.

The next important index, which assesses the quality of leather, is a strength of leather. The physical–mechanical properties of the fatliquored and dried leather are presented in Table 4.

The enzymatic pickling activated hide collagen and it became more reactive: it bonded more chromium and fatliquors, and dyes penetrated deeper. Unfortunately, the action of enzymes decreased the strength of the leather obtained from enzymatically pickled hide, comparing with that of the conventional one. The better opening up and distribution of microstructure during the enzymatic pickling led to higher relative elongation.

## 4. Conclusions

The employment of active-in-acidic-medium proteolytic enzymes in the pickling process led to an additional opening up of the derma structure and changed the properties of the hide. A level of the effect on collagen depended on the enzyme preparation used and its activity: the removal of collagenous proteins was 1.2–3.5 times higher during enzymatic pickling, compared with the conventional one. The intense opening of the dermal structure took place: it reduced the porosity of the hide dermis and increased the hide’s shrinkage temperature. Herewith, the strong effect by the enzyme in pickling did not cause any observable effect on the supermolecular structure of chromed leather, which could be reflected in IR-spectra.

It can be concluded that the enzymatically pickled pelt bonded more chromium and reached higher shrinkage temperature when chroming; dyes penetrated deeper, especially through the grain layer; such leather bonded more matter that is soluble in dichloromethane as well.

Unfortunately, the action of enzymes used in this study worsened the physical–mechanical properties of leather: the strength of the leather obtained from enzymatically pickled hide was lower than that of the conventional one. The first was characterised by a weaker grain layer and significantly higher relative elongation.

The action of the enzymes during pickling changed the properties of both pickled hide and chromed leather, so this could be applied to the leather processing if necessary. On the other hand, as some properties improved and others worsened during such a process, the application of every enzyme should be carefully investigated and optimized to produce leather with controlled properties.

## Figures and Tables

**Table 1:** Influence of enzyme preparation addition on pickled pelt properties.

Pickling Variant (EP Used and Its Amount)	Shrinkage Temperature, °C	Porosity, %
1 (Novo Bate WB 0.5%)	47.0 ± 0.6	61.4 ± 1.2
2 (Novo Bate WB 1%)	49.0 ± 0.6	58.8 ± 1.4
3 (Novo Bate WB 2%)	54.0 ± 0.9	55.2 ± 1.7
4 (Zime SB 1%)	46.0 ± 0.3	59.8 ± 0.9
5 (Zime SB 2%)	48.0 ± 0.8	55.7 ± 1.4
6 (Zime SB 3%)	56.0 ± 0.6	53.5 ± 1.0
7 (control—without EP)	48.0 ± 0.3	67.0 ± 2.1

**Table 2:** Influence of pickling method on chroming and chromed leather properties.

Pickling Variant (EP Used and Its Amount)	Chromium Exhaustion, % ^1^	Indexes of Chromed Leather		
		Cr_2_O_3_, % ^1^	Shrinkage Temperature, °C	Porosity, %
1 (Novo Bate WB 0.5%)	89.1 ± 0.2	2.63 ± 0.02	98.8 ± 1.4	58.2 ± 1.9
2 (Novo Bate WB 1%)	89.2 ± 0.1	2.72 ± 0.01	100.1 ± 0.8	58.0 ± 2.1
3 (Novo Bate WB 2%)	88.4 ± 0.4	2.60 ± 0.02	98.0 ± 0.6	56.4 ± 1.8
4 (Zime SB 1%)	80.4 ± 0.5	2.52 ± 0.02	102.0 ± 0.8	62.7 ± 1.4
5 (Zime SB 2%)	82.8 ± 0.3	2.62 ± 0.01	102.8 ± 1.2	58.4 ± 1.6
6 (Zime SB 3%)	88.0 ± 0.2	2.65 ± 0.03	104.5 ± 0.5	55.6 ± 1.3
7 (control—without EP)	80.2 ± 0.4	2.51 ± 0.02	97.4 ± 1.3	64.7 ± 1.8

^1^ Those percentages are by mass.

**Table 3:** Influence of pickling method on post-tanning processes and crust leather properties.

Pickling Variant (EP Used and Its Amount)	Dye Consumption, %	Depth of Dye Penetra-tion ^1^, %		Amount of Matter Soluble in Dichloromethane, %	Shrinkage Temperature, °C
		Flesh Side	Grain Side		
6 (Zime SB 3%)	62.2 ± 0.8	9.8 ± 0.2	12.2 ± 0.3	5.6 ± 0.2	104.2 ± 0.9
7 (control—without EP)	56.2 ± 0.7	8.3 ± 0.2	6.7 ± 0.2	4.8 ± 0.2	100.3 ± 1.1

^1^ Thickness of dyed leather samples was within 5.9–6.3 mm.

**Table 4:** Physical–mechanical properties of crust leather dependent on pickling method.

Pickling Variant (EP Used and Its Amount)	Relative Elongation of Leather at the Strain 10 N/mm^2^, %	Relative Elongation of Leather at the Break, %	Strain When Grain Layer Breaks, N/mm^2^	Tensile Strength of Leather, N/mm^2^
6 (Zime SB 3%)	58.1 ± 1.7	82.2 ± 2.1	16.0 ± 0.3	16.8 ± 0.2
7 (control—without EP)	40.4 ± 1.5	75.3 ± 2.0	18.9 ± 0.4	18.9 ± 0.4

## Data Availability

Data are contained within the article.
